# Estimating the Number of Patients Receiving Specialized Palliative Care Globally in 2017

**DOI:** 10.1016/j.jpainsymman.2020.09.036

**Published:** 2021-04

**Authors:** Stephen R. Connor, Carlos Centeno, Eduardo Garralda, David Clelland, David Clark

**Affiliations:** a Worldwide Hospice Palliative Care Alliance, London, United Kingdom; b ATLANTES Global Palliative Care Observatory, Institute for Culture and Society, University of Navarra, Pamplona, Navarra, Spain; c IdiSNA, Navarra Institute for Health Research, Pamplona, Navarra, Spain; d School of Interdisciplinary Studies, University of Glasgow-Dumfries, Scotland, United Kingdom

**Keywords:** Palliative care, hospice, mapping, global development, indicators

## Abstract

**Context:**

Palliative care is an emerging health-care service essential for every health-care system. Information on the current status of palliative care service delivery is needed to understand the gap between need for palliative care and current capacity to deliver.

**Objectives:**

To estimate the number of providers delivering palliative care worldwide and the patients they served in 2017.

**Methods:**

Estimates were obtained from a sample of countries from each World Bank income group using typical case purposive sampling methods. Reliable data from the United States and eight additional countries were used for the high-income group. For low- and middle-income countries (LMICs), to determine an estimate of the number of patients served, 30 countries representative of palliative care service delivery in each region and income group were surveyed.

**Results:**

Results from the mapping levels of palliative care development survey identified a total of approximately 25,000 palliative care service delivery teams globally. The total estimate of patients served in 2017 was approximately seven million.

**Conclusion:**

Significant disparities in palliative care access exist both by region and income group. The European and Pan-American regions had most while the Eastern Mediterranean, Southeast Asian, and African regions had least. Much more needs to be done to develop and deliver palliative care in LMICs where 80% of the need for palliative care exists. With about 70% of operating palliative care services in high-income countries and only 30% in LMICs, a major effort to develop palliative care in these settings is urgently needed.

## Key Message

Between 2011 and 2017, the number of palliative care services and those that were able to receive palliative care increased. Service providers went from approximately 16,000 to more than 25,000, and patients cared for from 3 to 7 million. However, this is only beginning to meet the large unmet need.

## Introduction

The Worldwide Hospice Palliative Care Alliance and the World Health Organization jointly published the Global Atlas of Palliative Care at the end of Life in 2014,^1^ which for the first time presented an estimate of the need for palliative care globally. This need was estimated at over 40 million patients at and before the end of life. More recently a Lancet Commission on Palliative Care and Pain Relief^2^ reached a higher estimate of the need for palliative care at over 60 million decedents and nondecedents. The ability to compare capacity to deliver palliative care against the need has been useful in describing the gap in unmet need for palliative care. This gap is expected to increase significantly in the coming decades. The need for palliative care, as measured by prevalence of serious health or illness-related suffering, is expected to increase 87% by the year 2060.^3^

Information on the current status of palliative care service delivery is needed to understand the gap between need for palliative care and current capacity to deliver. This gap has been described as meeting less than 10% of the need and less than 14% at the end of life.^4^

Palliative care is both a specialized area of health care and an important skill set across medical specialties and for primary care. The United Nations call for Universal Health Coverage includes promotion, prevention, treatment, rehabilitation, and palliative care signaling its importance as an essential element of health systems.^5^

Estimating the number of patients and families receiving palliative care globally faces significant challenges, not least of which is the lack of any system of national palliative care patient registries. However, there is no centralized global systematic reporting mechanism to track progress in access to this vital health-care service.

While palliative care is recognized as a medical specialty or subspecialty in over 30 countries, there is also lack of agreement as to the distinction between specialist and nonspecialist service delivery. The integration of palliative care into existing systems of medical specialization and the importance of embedding palliative care into national health systems has been emphasized. The need for generalized palliative care is particularly large, and it has been estimated that around two-third of patients needing palliative care could be cared for by primary palliative care services embedded in existing health-care systems.^6^ For this study, we were not able to measure all patients receiving palliative care by clinicians that were not members of a palliative care team.

The Global Atlas also presented an estimate of the number of patients receiving palliative care. This estimate of three million patients was based on an ongoing project, in a partnership between the Worldwide Hospice Palliative Care Alliance, the ATLANTES Observatory, and University of Glasgow, to map levels of palliative care development globally.^7^^,^^8^

The growth of palliative care provision in recent years has been relatively slow and uneven, with most countries reporting limited service delivery and integration into existing health-care systems. As of 2011, 73.6% of countries worldwide had little or no palliative care service delivery.^7^ More recently, an update of this mapping of levels of palliative care development has described some improvement, in that the proportion of countries worldwide with little or no service delivery has decreased to 64%,^9^ although much improvement is needed. This report is based in part on the results of this research. As part of the survey that is the basis of the mapping report, the number of palliative care services was collected for all participating countries. The number of services is the principal method used to derive estimates of the number of patients accessing palliative care services.

## Methods

As noted, this study is part of a larger longitudinal effort to measure the development of palliative care globally. The methods for the mapping study have been previously published.^10^ An online survey of experts in 198 countries generated 2017 data on 10 indicators of palliative care provision, linked to six categories of development. Factor analysis and discriminant analysis showed the validity of the categorization. Spearman correlation analyses assessed the relationship with World Bank Income Level, Human Development Index, and Universal Health Coverage to palliative care development. One of the 10 indicators was the number of palliative care services to population in the reporting country, where such services existed.

To estimate the number of patients receiving palliative care based on the number of service programs delivering palliative care, we needed to know the average number of people under care in any given service annually. As there is no global system for collecting this information, we had to ask countries represented in the survey to provide this information, particularly those in low- and middle-income settings.

By these means, estimates were obtained from a sample of countries from each World Bank income group (WBIG). Routinely collected data from the United States, Australia, and the United Kingdom on the number of patients under care were reliably available and used, along with six additional high-income country reports of estimated average numbers of patients served.^11^^,^^12^ For low- and middle-income countries (LMICs), to determine an estimate of the number of patients served, *typical case purposive sampling methods* were used to identify countries representative of palliative care service delivery in each region and income group. Random sampling was not feasible because of the limited number of countries that had any capacity for collecting data from services. A sample of 30 countries were chosen to include at least 30% of countries in each low- or middle-income group (Table 1).

**Table 1:** Estimation of Patients Attended per Service and per Year by World Bank Income Group

Sample			Average of Patients Attended per Service Annually
Income Group	Countries Sampled		
	Names	N Countries Sampled/N Countries in the Income Group (%)	
High income	Australia, Chile, Greece, Hungary, Japan, Singapore, Spain, UK, USA	9/52 (17)	324
Upper middle	Albania, Armenia, Brazil, Georgia, Iran, Jamaica, Kazakhstan, Lebanon, Malaysia, Mauritius, Russia, Serbia, South Africa	13/41 (32)	297
Lower middle	Bangladesh, Brazil, India, Kenya, Kyrgyzstan, Mongolia, Pakistan, Philippines, Tunisia, Ukraine	10/32 (31)	187
Low income	Burundi, Ethiopia, Ghana, Nepal, Rwanda, Sierra Leone, Tajikistan	7/21 (33)	133

This typical case purposive sampling method required key individuals in each country, mainly through the national palliative care association where one existed, to be contacted by email to determine and estimate the number of patients attended to per service provider per year. The typical number of patients attended to by a palliative care service, including home and/or inpatient unit, were then divided by the number of service providers in each WBIG to compute an average number of admissions per service for the WBIG category. The average number of admissions for each WBIG was then multiplied times the number of service programs providing palliative care in each country to calculate an estimate of the number of patients served globally.

## Results

A total of 146 countries out of 198 responded to our original survey with information that included numbers of services. Nine of the countries that responded to the survey indicated that no services existed. The countries that were contacted and from whom we collected data on average numbers of admissions are noted in Table 1. A total of 93 countries were in the upper-middle, lower-middle, or low-income country groups. As there were good estimates of the number of patients served in high-income countries, the focus was on determining the typical numbers served per service in the non–high-income groups.

### Specialized Palliative Care Services

Results from the mapping levels of palliative care development survey identified a total of approximately 25,000 palliative care service delivery teams globally. The number of services by World Health Organization region and WBIG can be seen in Table 2.

**Table 2:** Estimated Services Delivering Palliative Care Globally by Region and Income Group

Region or Group	N	%
WHO Region		
African	1085	4
Eastern Mediterranean	78	<1
European	9844	39
Americas	8605	34
Southeast Asia	800	3
Western Pacific	4987	19
Totals	25,399	100
World Bank Income Group		
High income	17,602	69
Upper middle	6450	25
Lower middle	1114	5
Low income	233	1
Totals	25,399	100

The largest numbers of palliative care services are found in the European and Pan-American regions while the fewest are found in the Eastern Mediterranean, Southeast Asian, and African regions. For the WBIGs, clearly the largest number of services by far are found in high-income countries followed by those in upper middle-income countries.

### Patients Receiving Palliative Care

The number of service providers was used to estimate the number of individuals receiving palliative care globally. In 2017, 1.49 million beneficiaries receive one or more days of hospice care provided by 4515 certified care providers in the United States or 330 patients per service.^10^ Based on this and eight other high-income countries surveyed, which had similar findings, each service provider served an average of 324 patients that year. Combining all the regions and groups shows significant disparities in access to palliative care both by region and income group. The total estimate of patients served in 2017 was approximately seven million (Table 3).

**Table 3:** Estimated Patients Receiving Palliative Care Globally by Region and Income Group

Region or Group	N	%
WHO Region		
African	231,689	3
Eastern Mediterranean	23,569	<1
European	2,943,900	43
Americas	2,202,152	31
Southeast Asia	187,214	3
Western Pacific	1,415,822	20.0
Totals	7,004,347	100.0
World Bank Income Group		
High income	4,812,380	69
Upper middle	1,935,548	27
Lower middle	227,009	3
Low income	29,410	1
Totals	7,004,347	100.0

Consistent with the availability of palliative care services, the largest numbers of patients receiving palliative care are found in the European and Pan-American regions while the fewest are found in the Eastern Mediterranean, Southeast Asian, and African regions. For the WBIGs, clearly the largest number of patients receive palliative care are in high-income countries (69%).

## Discussion

If every country had a palliative care registry, we would have more accurate numbers, but that is only available in high-income countries such as the United States, United Kingdom, and Australia. The number of patients receiving palliative care was estimated in 2011 to be around three million,^1^ while six years later, the 2017 estimate has climbed to about seven million. This is more than double the number served in 2011. Likewise, the number of palliative care services increased from approximately 16,000 to 25,000, a 64% increase. This represents a significant advance in access to palliative care globally; however, access to palliative care remains very limited.

The disparity between the need for palliative care and its availability is striking. Too often, palliative care is seen as a specialty that only well-resourced countries can afford. This is a wrong way to think as palliative care is an essential component of every health-care system and is included in the continuum of universal health care under goal three of the 2016 UN Sustainable Development Goals for the world.^13^

### Limitations

The definition of what constitutes a palliative care service is difficult to standardize and includes both home care teams, outpatient clinics, and inpatient services. Also, the distinction between specialized and generalized palliative care services is difficult to measure and needs further study. The services in this study may have consisted of either or both. The use of in-country experts can be criticized because of the subjective nature of responses and varying degrees of knowledge and expertise. Also, there may have been additional bias in that key informants may have overestimated the number of patients their programs actually served, which could have inflated the number of patients in each group resulting in the possibility that palliative care is even less accessible than estimated in this report.

The sample of countries used in this study may not accurately represent the delivery of palliative care in each WBIG as random sampling was not feasible because of lack of in-country record-keeping. The sample of high-income countries used only nine countries because of the fact that more reliable data on palliative care provision were available. However, World Bank Income grouping uses categorical variables to classify the income of countries and not continuous variables. None the less, this is the first effort to use enhanced methods to come up with an estimate of palliative care service provision globally that reflects differences in capacity to deliver palliative care in resource-limited settings.

This initial effort to measure access to palliative care services calls out the need for further research in this area including the need for creation of multivariate models for defining access to specialized palliative care services and the need for creation of data-collection and data-monitoring systems as well as further research on the impact of provision of palliative care.

## Conclusion

Much more needs to be done to develop and deliver palliative care in LMICs where nearly 80% of the need for palliative care exists. With about 70% of operating palliative care services in high-income countries and only 30% in LMICs, a major effort to develop palliative care in these settings is urgently needed.
